# Independent Validation of the SEND-PD and Correlation with the MDS-UPDRS Part IA

**DOI:** 10.1155/2014/260485

**Published:** 2014-10-23

**Authors:** Mayela Rodríguez-Violante, Amin Cervantes-Arriaga, Salvador Velázquez-Osuna, Rodrigo Llorens-Arenas, Humberto Calderón-Fajardo, Dan Piña-Fuentes, Pablo Martinez-Martin

**Affiliations:** ^1^Clinical Laboratory of Neurodegenerative Diseases, National Institute of Neurology and Neurosurgery, La Fama, 14269 Mexico City, Mexico; ^2^Movement Disorders Clinic, National Institute of Neurology and Neurosurgery, 14269 Mexico City, Mexico; ^3^National Center of Epidemiology and CIBERNED, Carlos III Institute of Health, 30201 Madrid, Spain

## Abstract

*Introduction*. Neuropsychiatric symptoms in Parkinson's disease can be assessed by the MDS-UPDRS part IA. The Scale for Evaluation of Neuropsychiatric Disorders in Parkinson's disease (SEND-PD) has been recently developed to assess the severity of some neuropsychiatric symptoms. The objective of this study is to compare the performance of the SEND-PD with the corresponding items of the MDS-UPDRS part IA. *Methods*. Patients with Parkinson's disease were evaluated using the MDS-UPDRS and the SEND-PD by independent raters. Partial SEND-PD and neuropsychiatric MDS-UPDRS part IA were constructed with equivalent items for comparison. *Results*. A total of 260 consecutive patients were included. Overall, 61.2% of the patients did not report any psychotic symptom and 83.5% did not report any ICD symptom. On the other hand, 78.5% of the patients did report at least one symptom related to apathy, depression, or anxiety. The partial SEND-PD score was 2.9 ± 3.1 (range from 0 to 16). The neuropsychiatric MDS-UPDRS part IA score was 2.9 ± 3 (range from 0 to 14). The correlation coefficient between corresponding items ranged from 0.67 to 0.98 and between both summary indexes was *r*_*s*_ = 0.93 (all, *P* < 0.001). *Conclusion*. A high association between equivalent items of the SEND-PD and the MDS-UPDRS was found.

## 1. Introduction

Parkinson's disease (PD) is a neurodegenerative disease characterized by both motor and nonmotor symptoms. Neuropsychiatric symptoms are amongst the most common nonmotor features of PD [1].

The International Parkinson and Movement Disorder Society has issued several recommendations for the use of clinical scales for the assessment of neuropsychiatric symptoms [2–5]. Unfortunately most of these instruments are time consuming, focused on a particular domain, and nonspecific for PD. Thus, a brief, comprehensive, and specific clinical instrument for neuropsychiatric symptoms screening and basic evaluation is of great value for neurologists.

The MDS-UPDRS part IA (Movement Disorders Society-Unified Parkinson's Disease Rating Scale) evaluates nonmotor symptoms in PD subjects and it has been shown to correlate with several validated scales for the nonmotor aspects of the disease [6, 7]. Neuropsychiatric and cognitive symptoms assessed by the MDS-UPDRS part IA include cognitive decline, hallucinations/psychosis, depressive mood, anxiety, apathy, and dopaminergic disregulation syndrome.

The Scale for Evaluation of Neuropsychiatric Disorders in Parkinson's Disease (SEND-PD) has been recently developed to assess the severity of some neuropsychiatric symptoms prominent in this disease [8]. The SEND-PD evaluates the presence and severity of psychotic symptoms, mood/apathy, and impulse control disorders (ICD).

The SEND-PD performance has not been compared with the MDS-UPDRS part IA. The objective of the present study is to analyze the convergent validity of the SEND-PD with the corresponding components of the MDS-UPDRS part IA.

## 2. Materials and Methods

We included consecutive PD patients attending the Movement Disorders Clinic at the Neurology and Neurosurgery National Institute in Mexico City. PD was diagnosed according to the Queen Square Brain Bank Criteria [9]. Demographic data including gender, age, and years of formal education were collected. Clinical data regarding age at PD onset, predominant symptoms at PD onset, and current treatment and dose were collected. Levodopa equivalent daily dose (LEDD) was calculated [10]. PD patients were evaluated by a neurologist with expertise in movement disorders using the Spanish version of the Movement Disorder Society-Unified Parkinson Disease Rating Scale (MDS-UPDRS) [11]. Disease severity was determined according to the Hoehn and Yahr staging [12].

Neuropsychiatric symptoms were assessed using the Spanish version of the SEND-PD by an independent rater blinded to the MDS-UPDRS score. The SEND-PD is 12-item scale designed to measure the presence and severity of neuropsychiatric symptoms. It uses a 5-point Likert scale (scores 0–4 for each question) to rate the severity of symptoms. Scores for psychotic symptoms range from 0 to 16; scores for mood/apathy ranges from 0 to 20; and scores for ICDs range from 0 to 12. In all cases a higher score indicates greater severity.

When patients showed a significant cognitive decline, or any other condition preventing them from filling the questionnaires, the information was obtained from patients and their caregivers or directly from the caregivers.

The local ethics and research committee approved this study. All participants provided written informed consent as determined by the local ethics committee.

### 2.1. Statistical Analysis

Demographic data were reported in terms of percentage and mean and standard deviation.

As main data were ordinal or did not fit normal distribution, nonparametric statistics were used. The floor and ceiling effect up to 15% [13] and the skewness between −1 and +1 [14] were considered acceptable. Internal consistency of the SEND-PD subscales was explored by corrected item-domain correlation and Cronbach's alpha. Values ≥0.30 [15] and ≥0.70 [16], respectively, were considered appropriate. Correlations between corresponding items of the SEND-PD and MDS-UPDRS part IA and between a “partial SEND-PD score” (items 4, 6, 8, 9, and 12) and the corresponding “partial MDS-UPDRS part IA Neuropsychiatric score” (items 1.2 to 1.6) were determined for this study. Spearman's correlation coefficient values between 0.30 and 0.70 were considered “moderate” and those >0.70 were considered “high” [17]. Internal validity of the SEND-PD was determined by interdomain correlations, with values from 0.30 to 0.70 deemed satisfactory [15].

## 3. Results

A total of 260 (53.1% male and 46.9% female) consecutive patients were included. The age of the sample (mean ± SD) was 62.4 ± 13.1 years and the mean years of schooling were 8.4 ± 5.2. Mean age of onset of disease was 55 ± 13.8 years (mean disease duration of 7.1 ± 5.4 years). Tremor-dominant form of Parkinson's disease was present in 60.8% of the patients. Regarding the Hoehn and Yahr stage, 69.6% of the sample had a mild disease (HY 1-2), 18.1% had a moderate disease (HY 3), and 12.3% had a severe disease. Regarding the antiparkinsonian treatment, 71.9% were on levodopa and 56.2% were on a dopamine agonist. Mean levodopa equivalent daily dosage was 584.2 ± 432.5 mg. Motor fluctuations were present in 13.6%, dyskinesia was in 24.4%, and freezing was in 25.2%. A total of 32 patients (12.3%) had dementia by clinical judgment.

Full SEND-PD and MDS-UPDRS were computable for all patients. The SEND-PD and MDS-UPDRS part I were responded by patients alone (76.5%), caregiver (1.2%), or by both patients and caregivers (22.3%).

MDS-UPDRS part I score was 12.1 ± 6.5; MDS-UPDRS part II score was 14.1 ± 9.8; MDS-UPDRS part III was 34.1 ± 18.8 and part IV was 2 ± 3.8.

The distribution of scores, acceptability, and internal consistency analyses of each SEND-PD subscale are shown in the Table 2. Overall, 61.2% of the patients did not report any psychotic symptom and 83.5% did not report any ICD symptom. On the other hand, 78.5% of the patients did report at least one symptom related to apathy, depression, or anxiety. The frequency of neuropsychiatric symptoms as reported in both the MDS-UPDRS part IA and SEND-PD is shown in Table 1. Power calculation for prevalence was carried out taking into account the lowest prevalence with any scale (0.031 for dopaminergic dysregulation in the SEND-PD). Considering a sample of 260 subjects, for a two-sided test to detect a proportion of 0.031 given a null mean of 0.01 and assuming a 5% significance level, the power is 0.80. There was no statistically significant difference in SEND-PD scores between men and women (5.1 ± 6.1 versus 5.4 ± 6, *P* = 0.67).

There was no relevant ceiling effect for the whole SEND-PD or any of its domains. Floor effect was 17.3% for the SEND-PD, being higher for the ICDs and psychotic symptoms subscales (83.5% and 61.2%, resp.). Cronbach's alpha was <0.70 only for the ICDs subscale (alpha = 0.53) and only one item (SEND-PD 12, dopaminergic drug abuse/addiction) showed corrected item-domain correlation <0.30 (*r* = 0.21).

Table 3 shows the convergent validity between the SEND-PD and other related measures of the MDS-UPDRS part IA. A very high correlation coefficient was found between hallucinations item 4 of the SEND-PD and the corresponding item 1.2 of the MDS-UPDRS part IA (*r*_*s*_ = 0.92), as well as between depression and anxiety related items (*r*_*s*_ = 0.96 and 0.98, resp.). Dopaminergic dysregulation syndrome item also had a high correlation but it should be mentioned that the frequency of this disorder was very low. Apathy was the item showing the lower correlation, although it was still moderate.

The partial SEND-PD score was 2.9 ± 3.1 (range from 0 to 16). The neuropsychiatric MDS-UPDRS part IA score was 2.9 ± 3 (range from 0 to 14). The correlation coefficient between both resumed indexes was high (*r*_*s*_ = 0.93, *P* < 0.001). Concerning the internal validity, correlation coefficient values ranged from 0.38 (psychotic symptoms with mood/apathy) to 0.27 (mood/apathy with ICDs).

## 4. Discussion

With the growing understanding of neuropsychiatric symptoms and other nonmotor manifestations in Parkinson's disease (PD), this entity is no longer conceptualized as a pure motor disorder. Among these symptoms, impulse control disorders, psychosis, and depression/apathy stand out due to the important detrimental consequences. Impulse control disorders are characterized by a failure to resist an impulse to perform an activity that is harmful to the person or to others, due to its excessive nature [18]. ICDs may raise severe social, economic, and legal issues for both patients and their caregivers. The frequency of ICDs has been reported to be 13.6% [19]. The assessment and diagnosis of an ICD usually require the use of extended and time-consuming clinical scales such as the Minnesota Impulsive Disorders Interview. The Questionnaire for Impulsive-Compulsive Disorders has been validated for PD as a screening instrument [20] but may also result in being time consuming when applied along with other neuropsychiatric scales. The MDS-UPDRS has only one item for assessing ICDs, specifically termed as dopaminergic dysregulation syndrome.

Hallucinations and psychotic symptoms present in 30%–40% of patients with PD [21, 22]. In regard to psychotic symptoms no PD-specific scale has been completely validated. The presence of hallucinations and other psychotic symptoms is usually evaluated through the use of instruments designed for schizophrenic disorders such as the Positive and Negative Symptom Scale or the Brief Psychiatric Rating Scale [4].

The prevalence of apathy has been reported to be between 17% and 51% [23], while depression is found in around 30 to 40% [24, 25]. Several scales have been recommended for screening and diagnosis of apathy and depression in patients with Parkinson's disease [2, 3]; nevertheless most of them are not suitable for routinely screening in an outpatient clinic setting.

The SEND-PD scale was designed to evaluate psychotic symptoms, mood, apathy, and impulse control disorders in a simple and relatively fast way [8].

In the present study, data quality was adequate and skewness values were slightly higher than the accepted upper limit. This fact is consistent with the floor effect subsequent to the high proportion of patients who did not experience the symptoms included in some domains. A high floor effect was also reported in the first validation study of the scale [8]. Nevertheless, the floor effect found in our sample was approximately 10% higher in all the subscales and probably reflects the lower global disease severity in our sample. Internal consistency index (Cronbach's alpha) for ICDs subscale was below the adequacy criterion. Cronbach's alpha is influenced by the distribution of scores in the sample and the number of items in the scale, two factors that can explain the relatively low value of this index for the ICD dimension (three items). In addition to differences in the assessed constructs, the low prevalence of ICDs also can explain the loose association of this dimension with the other domains composing the SEND-PD.

The SEND-PD was originally validated using the Scales for Outcomes in Parkinson's Disease-Psychiatric Complications (SCOPA-PC), while the motor state was evaluated with the Scales for Outcomes in Parkinson's Disease-Motor (SCOPA-Motor). The MDS-UPDRS, specifically part IA, has been validated using the Hamilton Depression Scale, Hospital Anxiety and Depression Scale, Lille Apathy Rating Scale, and Parkinson's Psychosis Rating Scale, among other scales [6], as well as the Nonmotor Symptoms Scale [7]. In our study, high correlation coefficients were found between SEND-PD scores and similar items of the MDS-UPDRS. Apathy was the only item showing a moderate correlation. Differences on the apathy item construct between scales may partially explain this finding. For instance, item 1.5 from the MDS-UPDRS IA explicitly assesses the performance of daily activities as well as the social interactions. On the other hand, the item from the SEND-PD is focused exclusively on initiating, participating, or finishing tasks or activities.

Moreover, the correlation between the partial SEND-PD and neuropsychiatric MDS-UPDRS part IA scores was extremely high (>0.90), demonstrating almost equivalence between both measures.

Our study has limitations. Since the study was carried out in a tertiary care setting, a referral bias was present resulting in an underrepresentation of patients with a more severe disease. Thus, extrapolation of our findings to this population should be taken with caution and comparison with the results of the original study is ballasted by differences in PD severity between samples. Another limitation is that comparison with a gold standard evaluation was not performed; thus sensitivity and specificity could not be calculated. Nevertheless, both the MDS-UPDRS part IA and the SEND-PD have been previously validated with such instruments and proved to be adequate for screening purposes. Finally, it should be mentioned that while the MDS-UPDRS part IA has one item for cognitive decline assessment, the lack of such domain on the SEND-PD did not allow any comparison.

## 5. Conclusion

In conclusion, the present study confirms the data from the original study and demonstrates that the SEND-PD, as a whole, is an acceptable, consistent, and valid assessment for presence and estimation of severity of the neuropsychiatric disorders associated with PD. In addition, findings indicate that corresponding items and scores of the SEND-PD and MDS-UPDRS part IA are strongly associated, reinforcing the satisfactory convergent validity from previous studies for both scales. For research purposes, the choice of one scale over the other will depend greatly on the primary endpoint, time availability, and personal preferences. The SEND-PD change over time has yet to be studied. For clinical use, both scales can be considered equivalent for assessing hallucinations, psychosis, depression, anxiety, apathy, and dopaminergic dysregulation syndrome.

## Figures and Tables

**Table 1:** Frequency∗ of neuropsychiatric symptoms assessed by the MDS-UPDRS part IA and SEND-PD.

	MDS-UPDRS IA	SEND-PD	*P*
Hallucinations/psychosis	52 (20%)	49 (18.8%)	0.82
Depression	158 (60.8%)	160 (61.5%)	0.92
Anxiety	114 (43.8%)	116 (44.6%)	0.92
Apathy	95 (36.5%)	86 (33.1%)	0.46
Dopamine dysregulation syndrome	9 (3.5%)	8 (3.1%)	0.81

^*^Frequency of responses >0 for each item.

**Table 2:** Clinimetric properties of SEND-PD subscales.

	SEND-PD subscales		
	Psychotic symptoms	Mood/apathy	Impulse control disorders
Mean ± SD	1.1 ± 2.1	3.8 ± 4	0.4 ± 1.1
Median (IR)	1	5	0
Range	0–14	0–19	0–8
Skewness	3.3	1.4	3.9
Cronbach's *α*	0.73	0.82	0.52
Corrected item-total correlation	0.40–0.68	0.59–0.64	0.21–0.46
Floor effect (%)	61.2%	21.5%	83.5%
Ceiling effect (%)	0%	0%	0%

SD: standard deviation. IR: interquartile range.

**Table 3:** Correlation between SEND-PD and MDS-UPDRS part IA related items and domains.

		MDS-UPDRS 1A				
		Item 1.2 (hallucinations/psychosis)	Item 1.3 (depressive mood)	Item 1.4 (anxiety)	Item 1.5 (apathy)	Item 1.6 (dopaminergic dysregulation)
SEND-PD	Item 4 (hallucinations)	**0.92**	0.24	0.25	0.09	0.13
	Total subscale 1 (psychotic symptoms)	**0.64**	0.30	0.36	0.21	0.19
	Item 6 (apathy)	0.23	0.44	0.34	**0.67**	−0.01
	Item 8 (depression)	0.20	**0.96**	0.47	0.37	0.18
	Item 9 (anxiety)	0.25	0.48	**0.98**	0.31	0.23
	Total subscale 2 (mood/apathy)	0.26	**0.75**	**0.71**	**0.63**	0.19
	Item 12 (abuse of dopaminergic drugs)	0.20	0.17	0.21	−0.01	**0.83**
